# Increasing prediction accuracy of pathogenic staging by sample augmentation with a GAN

**DOI:** 10.1371/journal.pone.0250458

**Published:** 2021-04-27

**Authors:** ChangHyuk Kwon, Sangjin Park, Soohyun Ko, Jaegyoon Ahn

**Affiliations:** 1 Center for Bioinformatics, EONE Laboratories, Incheon, The Republic of Korea; 2 Department of Computer Science and Engineering, Incheon National University, Incheon, The Republic of Korea; Politechnika Krakowska im Tadeusza Kosciuszki, POLAND

## Abstract

Accurate prediction of cancer stage is important in that it enables more appropriate treatment for patients with cancer. Many measures or methods have been proposed for more accurate prediction of cancer stage, but recently, machine learning, especially deep learning-based methods have been receiving increasing attention, mostly owing to their good prediction accuracy in many applications. Machine learning methods can be applied to high throughput DNA mutation or RNA expression data to predict cancer stage. However, because the number of genes or markers generally exceeds 10,000, a considerable number of data samples is required to guarantee high prediction accuracy. To solve this problem of a small number of clinical samples, we used a Generative Adversarial Networks (GANs) to augment the samples. Because GANs are not effective with whole genes, we first selected significant genes using DNA mutation data and random forest feature ranking. Next, RNA expression data for selected genes were expanded using GANs. We compared the classification accuracies using original dataset and expanded datasets generated by proposed and existing methods, using random forest, Deep Neural Networks (DNNs), and 1-Dimensional Convolutional Neural Networks (1DCNN). When using the 1DCNN, the F1 score of GAN5 (a 5-fold increase in data) was improved by 39% in relation to the original data. Moreover, the results using only 30% of the data were better than those using all of the data. Our attempt is the first to use GAN for augmentation using numeric data for both DNA and RNA. The augmented datasets obtained using the proposed method demonstrated significantly increased classification accuracy for most cases. By using GAN and 1DCNN in the prediction of cancer stage, we confirmed that good results can be obtained even with small amounts of samples, and it is expected that a great deal of the cost and time required to obtain clinical samples will be reduced. The proposed sample augmentation method could also be applied for other purposes, such as prognostic prediction or cancer classification.

## Introduction

Correct prediction of cancer stage is beneficial because it can help medical doctors determine more appropriate treatment for patients with cancer. For example, doctors can use staging information to determine type of surgery to perform, or whether chemotherapy or radiation therapy is required.

Numerous measures or methods have been proposed for accurate prediction of cancer stage, and one of the most widely used for cancer stage prediction is the Tumor, Node, and Metastasis (TNM) staging system developed by the American Joint Committee on Cancer (AJCC). TNM is a clinically useful staging system for cancers of almost every anatomic site and histology. From the 7^th^ edition of the AJCC Cancer Staging Manual to the most recent 8^th^ edition, few changes may be observed with respect to some cancers [1, 2], but in other cancer types, such as lung, gastric, and breast cancer [3–6] numerous changes are present in the criteria for prediction of cancer stage. These changes in the criteria may cause confusion in patient treatment.

Recently, alternative methods to predict cancer stage with additional clinical information or genomic information have been proposed. These methods, for the most part, adopt machine learning techniques to increase prediction accuracy. The machine learning methods used include Random Forest (RF) [7, 8], Support Vector Machine (SVM) [9], Naïve Bayes (NB) [9, 10], J48 Decision Tree [11], Logistic Regression [10, 11], Neural Network (NN) [12], and Neuro-Fuzzy Model [13]. In many cases, these methods showed better performance than the TNM staging system. For example, the Neuro-Fuzzy computational intelligence model [13] classified the pathological stage of patients with prostate cancer using data from The Cancer Genome Atlas (TCGA) [14], and compared these results with results using the AJCC pTNM (Pathological Tumor-Node-Metastasis) Staging Nomogram, as well as other machine learning methods such as Artificial Neural Network (ANN) or SVM, and found fewer false positives than the number achieved with AJCC or other machine learning models.

However, most of this studies used machine learning methods on a relatively small number of samples. machine learning methods generally require a substantial number of samples to ensure high predictive power. To overcome this limitation of a small sample size, many sample augmentation methods have been developed. The Synthetic Minority Oversampling Technique (SMOTE) [15, 16] was primarily developed to oversample a small number of samples, and has additionally shown its ability to convert highly imbalanced data into balanced data. Since 2012, the technique of deep learning has been applied in many fields, and the application of Denoising Autoencoder (DA) [17] solved the problem of insufficient training samples by expanding small gene expression data. Generative Adversarial Networks (GANs) [18] can be used to generate synthetic samples. GANs and their variations are widely used to synthesize images, but they can be also used to generate table type numerical data, as well as tabular data such as medical or educational records. TableGAN [19] shows that fake tables that are statistically similar to the original table are synthesized using GANs using four real world datasets in four different domains to solve the security problems required when sharing or delivering the public or partners’ data. Tabular GAN (TGAN) [20] shows the GANs model by applying Long Short-term Memory (LSTM) with attention to generate column-by-column data using tabular datasets of three mixed variable types.

In this study, we also used GANs to oversample small number of mRNA expression samples. GANs are difficult to use for data with a small sample size, especially when the number of features (genes) exceeds 10,000. To solve this problem, we first selected 300–800 genes depending on cancer types using DNA mutation data and RF. We synthesized the expression profiles of selected genes by applying GANs to gene expression of twelve cancer types including STAD (Stomach adenocarcinoma), BRCA (Breast invasive carcinoma), HNSC (Head and Neck squamous cell carcinoma), KIRC (Kidney renal clear cell carcinoma), KIRP (Kidney renal papillary cell carcinoma), LUAD (Lung Adenocarcinoma), THCA (Thyroid carcinoma), READ (Rectal adenocarcinoma), ESCA (Esophageal carcinoma), KICH (Kidney Chromophobe), LIHC (Liver hepatocellular carcinoma), and LUSC (Lung squamous cell carcinoma) from the TCGA database [14]. We then classified the cancer stage of augmented data using three classification methods. Comparison of the original data and augmented data obtained using existing sample augmentation methods allowed us to confirm that the prediction accuracy of cancer stage was significantly improved.

This paper is organized as follows. In the Materials and Methods Section, we first describe data used for the experiment, selected features, and normalization algorithm. Then, the sample augmentation method using GAN and three classification algorithms are described. In the Results Section, we describe the characteristics of the augmented sample, and compare the effects of the five known algorithms and four GAN series that we implemented. We also verify whether our method is effective for small samples, and evaluate the importance of the selected genes. In the Discussion Section, we compare the selection criteria of our experiment with the results of other groups, and mention various fields in which our method could be applied.

● We use feature selection based on DNA mutation data and GAN for augmentation of mRNA expression data to increase the accuracy of our cancer-stage classification.
● The augmented datasets obtained using the proposed method demonstrate significant increase in the classification accuracy.
● By using GAN and 1DCNN in the prediction of cancer stage, good results are obtained even with a small amount of sample.

## Materials and methods

### Data preparation and feature selection

We downloaded mRNA and DNA mutation data from the TCGA database [14] of twelve cancer types, STAD, BRCA, HNSC, KIRC, KIRP, LUAD, THCA, READ, ESCA, KICH, LIHC, and LUSC, which have at least twelve samples for all four stages. From downloaded data, only samples of which DNA and RNA IDs are matched and stage information exists were selected. Specific information regarding the data is provided in Table 1.

**Table 1:** Number of samples and features.

Type	#samples (I/II/III/IV)	#samples	All genes	Selected genes	Type	#samples (I/II/III/IV)	#samples	All genes	Selected genes
STAD	52/ 111/ 154/ 39	356	19,969	431	KIRP	137/ 19/ 42/ 13	211	19,216	773
BRCA	158/ 548/ 218/ 18	942	19,738	359	LUAD	262/ 119/ 77/ 26	484	19,648	360
HNSC	25/ 67/ 71/ 233	396	19,132	513	THCA	248/ 47/ 96/ 48	439	19,239	775
KIRC	250/ 51/ 100/ 70	471	19,216	649	READ	12/ 24/ 29/ 12	77	19,096	769
ESCA	18/ 77/ 55/ 9	159	19,629	717	KICH	18/ 24/ 13/ 5	60	19,216	711
LIHC	135/ 62/ 73/ 3	270	18,764	347	LUSC	237/ 158/ 81/ 4	480	19,648	397

As the feature space is too big compared to the number of samples for training the proposed model, we selected the most important features (= genes) for each dataset. RF classifier [7, 21], which showed the best performance, was used to select ranking genes using DNA mutation data. Through iterative experiments, we selected the p-value threshold as 0.004. The selected number of the most important features selected are shown in Table 1, and the list of genes is provided in S1 Table.

Finally, matched mRNA data with selected genes were normalized using ComBat [22] to correct batch effects.

### Sample augmentation and classification algorithm

The Generative Adversarial Networks (GANs) are composed of the generator and discriminator, which are trained in parallel. Typically, the generative network learns to map from a latent space to a data distribution of interest, while the discriminative network distinguishes candidates produced by the generator from the true data distribution.

In this study, we used a GANs to augment mRNA samples. When images are generated using GANs, random values are input to the generator. In our case, random values from a normal distribution with mean and standard deviation of training mRNA data are fed into the generator. The training data are 70% of the entire data, selected at random. We used one hidden layer with 256 neurons for both a generator and a discriminator with reference to the previous study [23] and the randomly synthesized data and real data are judged to be real or fake in the discriminator, and learned repeatedly. The number of epochs used varies from 900 to 1,100 depending on the cancer type.

After the generator is trained, we generate *n* (= number of training samples) samples (GAN1), *n* * 20 samples (GAN20), and *n* * 100 samples (GAN100), using the trained generator, with the latent space generated by mean and standard deviation values that were used to train the generator. The mean and standard deviation created to make latent space in the Training Step are stored at a global variable and selected randomly as argument of the Generating Step. The ratio of stages is kept for augmented samples. Augmented samples are used as training data for classification of cancer stage.

We used three types of classifiers, 1DCNN [24], DNNs, and RF [7]. 1DCNN has been proposed to process 1-dimentional spectral channels. The 1DCNN we used for this study consists of two convolution layers. In this study, 20 and 40 filters with kernel size of 5 were used for first and second convolution layers, respectively. For both layers, size of pool is two and Relu is used for activation function. After the convolution step, the flattening process is performed, and flattened values are fed into the hidden layer of size 64. Activation function is Relu, optimizer is Adam, batch size is 32, and number of epochs is 1,000. For DNNs, we used three hidden layers of size 64, 32, and 4. Activation functions used are Relu for hidden layers and Softmax final layer. Adam is used for optimizer. We used the RandomForestClassifier module of scikit-learn (version 0.23.2) in python (version 3.5.2). The number of trees in the forest (n_estimators) is 100, the oob_score (whether to use out-of-bag samples to estimate the generalization accuracy) is true, and the random_state (random value) is 123456. We tried varying the number of n_estimators (70, 100, and 130), and adopted 100 according to S3 Table.

**Fig 1:**
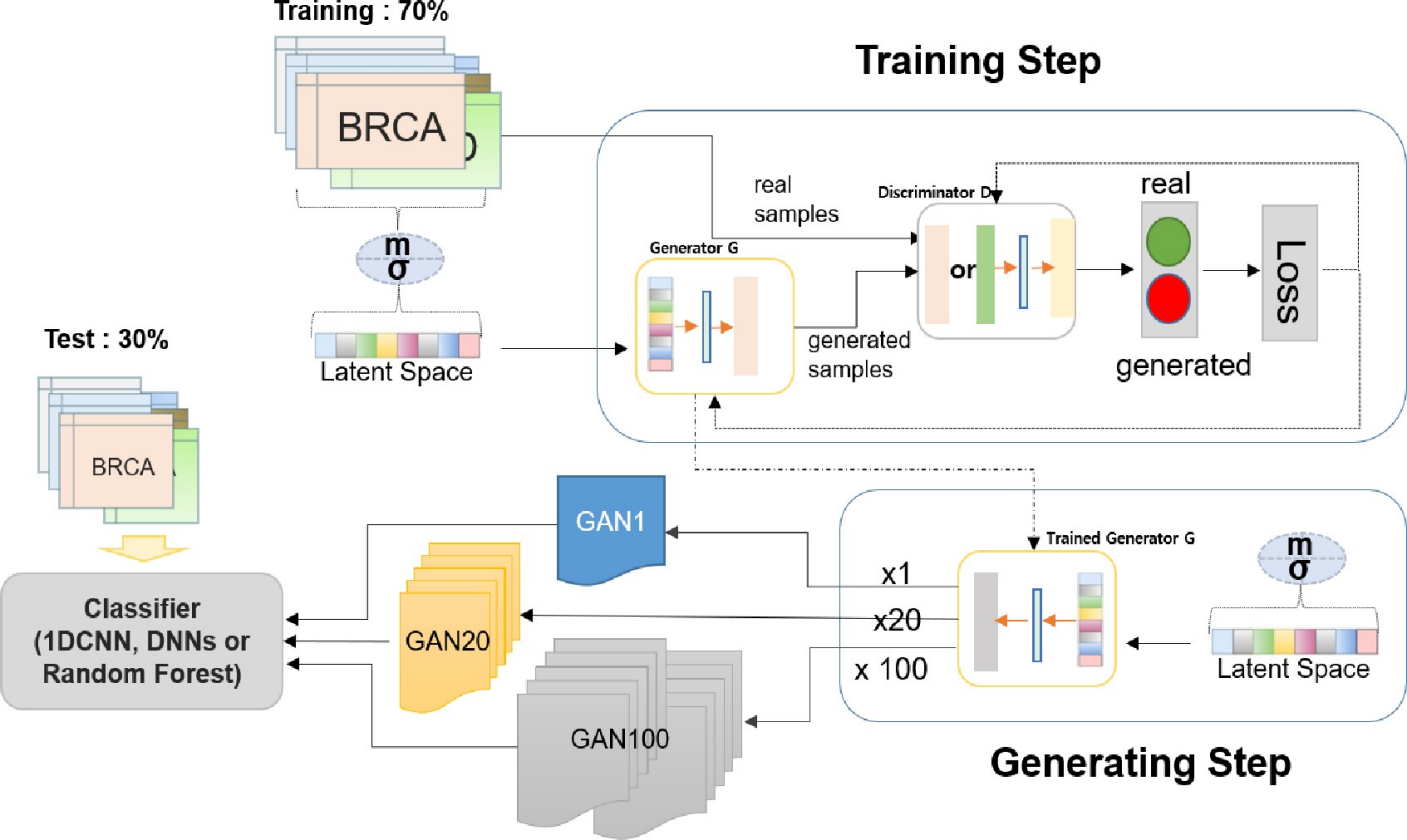
Overview of sample augmentation using GANs and classification of stages using augmented samples.

Finally, these classifiers were evaluated using the remaining 30% of the entire sample. The steps described above form one cycle, and are illustrated in (Fig 1).

## Results

### Characteristics of augmented samples

As mentioned in detail in the methods, we augmented samples by constructing GANs composed of components of a Generating Step and Training Step (as shown in Fig 1). These augmented samples were used for training three classifiers and the remaining 30% of the original data were classified using the classifiers. To characterize the augmented samples and to confirm the possibility that augmented samples can be effectively used for cancer stage classification, we performed principal component analysis (PCA) for the original dataset and the augmented dataset.

**Fig 2:**
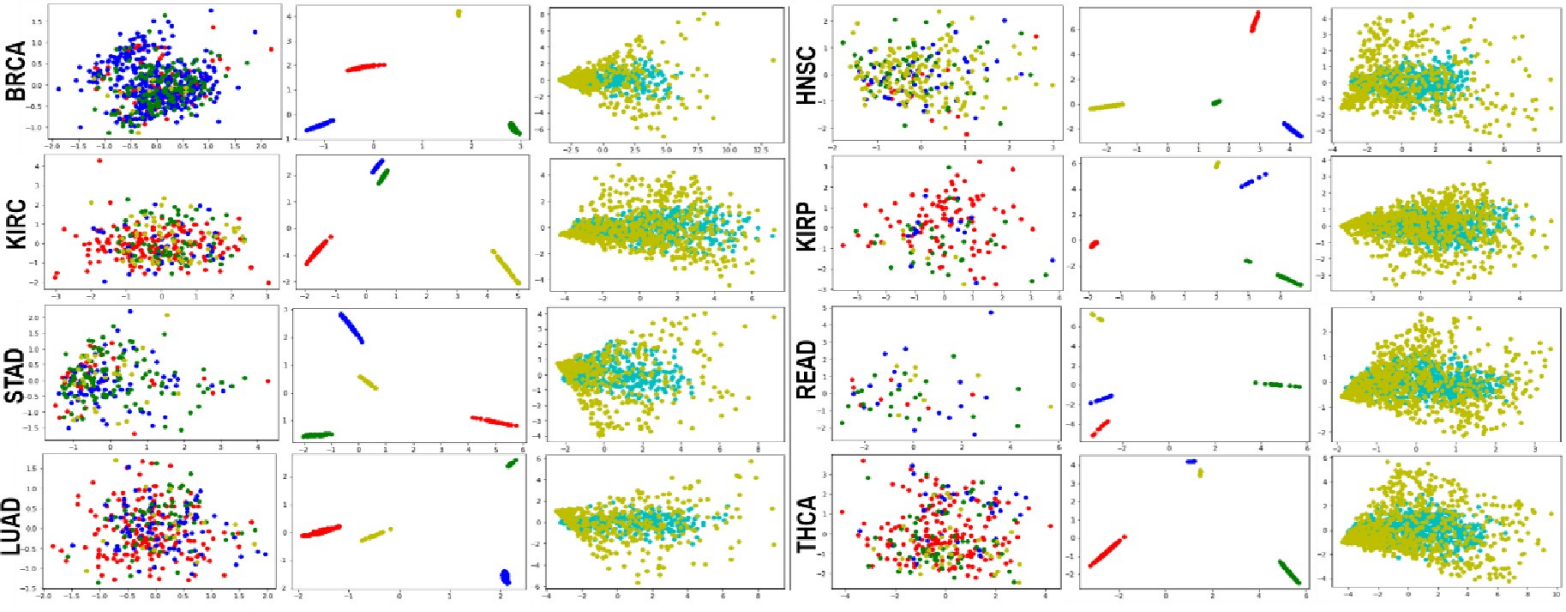
PCA plots for original and generated data for each cancer type. First and second columns are PCA plots of original and generated data samples (GAN1), respectively (stage1: red, stage2: blue, stage3: green, stage4: yellow). Third column is PCA plot for genes of original (cyan) and generated data (GAN1, yellow).

The first column of (Fig 2) shows PCA plots for the original dataset for eight cancer types, and we can see that the stages are not distinguished. However, we can see that the stages are clearly distinguished for GAN1 data. These results imply that augmented samples have different characteristics for each stage. The differences in the augmented samples are not the result of changes in gene expression patterns, however, as we can see that the distribution of gene expression is not very different between the original and augmented data, as shown in the third column in (Fig 2).

### The effect of sample augmentation

To evaluate the effect of sample augmentation, we created three classification models (using RF, 1DCNN, and DNNs) for each of the nine datasets. The nine datasets are 1) original dataset (Ori), 2) original dataset with selected features only (FS), 3) synthesized data with mean and standard deviation (MS), 4) synthesized data using SMOTE [16] (SMOTE), 5) synthesized data using DA [17] (DA), 6) GAN1, 7) GAN5, 8) GAN20, and 9) GAN100. All experiments using twelve datasets and three classifiers were repeated 10 times.

Features of FS data are selected from DNA mutation data using RF classifier, and are the same as those used to create GAN1, GAN5, GAN20, and GAN100. MS is randomly generated samples using mean and standard deviation/2 of training samples of each stage. SMOTE data is generated using a basic algorithm in SMOTE [16].

**Fig 3:**
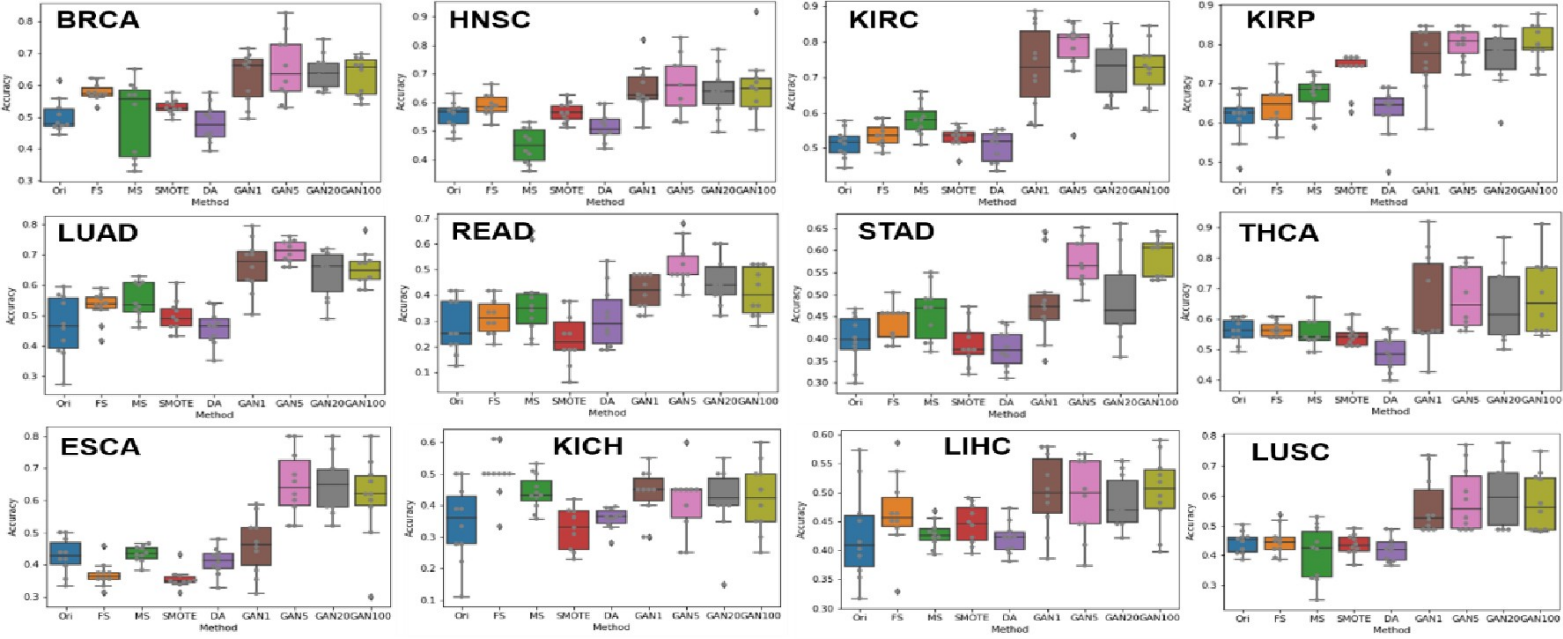
Comparing classification accuracy using 1DCNN with different datasets. Ori: Original data, FS: data with selected features, MS: randomly generated data using the mean and standard deviation of the FS data, DA: Denoising Autoencoder. P-values of t-test between three GAN results (GAN1, GAN5, GAN20 and GAN100) and five comparison results (Ori, FS, MS, SMOTE and DA) are given in S2 Table.

SMOTE is proposed to handle imbalanced data. For example, if SMOTE is run using 657 (110/383/152/12) training samples of BRCA, it generates 1,532 (383/383/383/383) samples. DA data is generated using a Denoising Autoencoder [17]. DA uses the denoising method to extract features that obtain useful structure in the input distribution and eventually generate gene expression data. Given n samples and m features, DA generates n * floor (m / 5) + n samples (floor (x) returns a largest integer not greater than x). For example, breast cancer has 659 training samples and 19,738 features, so 2,601,732 samples are generated. In (Fig 3), we can see that GAN1, GAN5, GAN20, and GAN100 show an increase over compared datasets. S2 Table shows that most of the p-values from t-tests between GAN and comparison results are < 0.05. In particular, all GAN5 showed significantly increased accuracy and most GAN20 datasets showed good accuracy.

We can also see that the accuracies of FS increased up to 9% compared to Ori, and the error bars are narrowed except in the case of KIRP. In particular, the accuracy was 0.48 for the 19,738 gene features in BRCA, but increased to 0.57 using a selected 359 features. These results show the effect of gene selection using DNA mutation data.

Next, we compared three classifiers, 1DCNN, DNN, and RF. Tables 2–13 show the accuracy and F1 score for each dataset and for each cancer type. Tables 2–13 also show that GAN1, GAN5, GAN20, and GAN100 demonstrate better predictive performance, regardless of classifier. Overall, 1DCNN and DNN showed good results and RF showed a poor F1 score.

**Table 2:** Comparison result of three classifiers for LUAD (mean±sdv).

	1DCNN		RF		DNN	
	Acc	F1	Acc	F1	Acc	F1
Ori	0.47±0.10	0.33±0.12	0.53±0.04	0.42±0.05	0.44±0.09	0.42±0.05
FS	0.53±0.05	0.40±0.04	0.52±0.02	0.41±0.04	0.39±0.04	0.40±0.02
MS	0.55±0.06	0.45±0.08	0.53±0.06	0.32±0.09	0.48±0.05	0.35±0.07
SM	0.50±0.05	0.40±0.03	0.52±0.06	0.45±0.07	0.41±0.11	0.34±0.09
DA	0.46±0.08	0.40±0.03	0.43±0.07	0.61±0.02	0.46±0.06	0.41±0.03
G1	0.66±0.09	0.65±0.09	0.67±0.06	0.45±0.08	0.74±0.05	0.74±0.04
G5	0.71±0.03	0.71±0.04	0.63±0.07	0.43±0.09	0.74±0.04	0.72±0.05
G20	0.64±0.08	0.64±0.07	0.61±0.07	0.41±0.07	0.73±0.07	0.77±0.02
G100	0.65±0.06	0.65±0.05	0.59±0.07	0.40±0.06	0.74±0.05	0.70±0.07

**Table 3:** Comparison result of three classifiers for KIRC (mean±sdv).

	1DCNN		RF		DNN	
	F1	Acc	F1	Acc	F1	
Ori	0.51±0.04	0.39±0.11	0.56±0.03	0.46±0.04	0.46±0.11	0.48±0.05
FS	0.54±0.03	0.39±0.05	0.55±0.03	0.46±0.04	0.41±0.08	0.45±0.04
MS	0.58±0.05	0.46±0.07	0.41±0.11	0.29±0.10	0.42±0.12	0.33±0.10
SM	0.53±0.03	0.36±0.02	0.56±0.03	0.49±0.04	0.42±0.15	0.27±0.12
DA	0.50±0.04	0.45±0.08	0.48±0.09	0.66±0.03	0.51±0.04	0.50±0.07
G1	0.73±0.12	0.72±0.12	0.71±0.04	0.59±0.05	0.62±0.15	0.68±0.14
G5	0.78±0.09	0.78±0.06	0.70±0.02	0.58±0.06	0.65±0.07	0.72±0.07
G20	0.72±0.08	0.71±0.08	0.71±0.03	0.59±0.06	0.58±0.17	0.68±0.13
G100	0.72±0.08	0.72±0.07	0.72±0.05	0.60±0.07	0.63±0.13	0.71±0.10

**Table 4:** Comparison result of three classifiers for STAD (mean±sdv).

	1DCNN		RF		DNN	
	Acc	F1	Acc	F1	Acc	F1
Ori	0.40±0.06	0.30±0.06	0.43±0.05	0.37±0.05	0.35±0.04	0.36±0.03
FS	0.44±0.04	0.28±0.08	0.43±0.05	0.37±0.05	0.32±0.03	0.33±0.05
MS	0.46±0.06	0.38±0.07	0.39±0.06	0.29±0.06	0.37±0.10	0.29±0.11
SM	0.39±0.05	0.27±0.07	0.40±0.04	0.37±0.06	0.25±0.05	0.13±0.02
DA	0.39±0.06	0.34±0.06	0.36±0.06	0.52±0.06	0.38±0.04	0.37±0.03
G1	0.48±0.09	0.50±0.07	0.53±0.06	0.41±0.09	0.55±0.13	0.59±0.08
G5	0.57±0.05	0.57±0.04	0.47±0.09	0.37±0.08	0.62±0.09	0.60±0.11
G20	0.49±0.10	0.51±0.08	0.48±0.11	0.38±0.02	0.53±0.12	0.63±0.09
G100	0.59±0.04	0.52±0.08	0.47±0.07	0.36±0.07	0.57±0.13	0.59±0.10

**Table 5:** Comparison result of three classifiers for READ (mean±sdv).

	1DCNN		RF		DNN	
	Acc	F1	Acc	F1	Acc	F1
Ori	0.28±0.11	0.17±0.10	0.30±0.06	0.24±0.07	0.30±0.06	0.30±0.14
FS	0.32±0.07	0.20±0.13	0.33±0.09	0.27±0.10	0.28±0.10	0.25±0.07
MS	0.38±0.15	0.26±0.14	0.26±0.07	0.18±0.08	0.33±0.07	0.24±0.06
SM	0.23±0.10	0.18±0.09	0.25±0.10	0.22±0.14	0.36±0.08	0.23±0.07
DA	0.32±0.14	0.32±0.14	0.35±0.07	0.43±0.10	0.31±0.12	0.32±0.10
G1	0.41±0.07	0.31±0.09	0.47±0.07	0.27±0.06	0.41±0.09	0.35±0.06
G5	0.52±0.08	0.39±0.03	0.47±0.07	0.29±0.05	0.42±0.04	0.36±0.04
G20	0.46±0.09	0.36±0.09	0.47±0.08	0.28±0.07	0.40±0.08	0.38±0.05
G100	0.41±0.09	0.33±0.10	0.46±0.08	0.28±0.06	0.41±0.10	0.36±0.07

**Table 6:** Comparison result of three classifiers for KIRP (mean±sdv).

	1DCNN		RF		DNN	
	Acc	F1	Acc	F1	Acc	F1
Ori	0.61±0.06	0.52±0.16	0.71±0.05	0.65±0.06	0.63±0.07	0.63±0.07
FS	0.65±0.06	0.53±0.19	0.71±0.05	0.63±0.07	0.59±0.09	0.60±0.07
MS	0.67±0.04	0.56±0.05	0.60±0.10	0.47±0.12	0.56±0.07	0.46±0.08
SM	0.73±0.05	0.62±0.07	0.72±0.06	0.68±0.07	0.57±0.05	0.46±0.07
DA	0.63±0.07	0.61±0.03	0.56±0.10	0.72±0.09	0.63±0.06	0.61±0.05
G1	0.76±0.08	0.71±0.09	0.75±0.06	0.41±0.09	0.77±0.05	0.72±0.05
G5	0.80±0.04	0.80±0.03	0.74±0.06	0.39±0.08	0.77±0.11	0.74±0.11
G20	0.77±0.08	0.72±0.07	0.73±0.02	0.37±0.06	0.79±0.07	0.74±0.04
G100	0.80±0.05	0.77±0.05	0.73±0.08	0.48±0.04	0.75±0.17	0.74±0.06

**Table 7:** Comparison result of three classifiers for HNSC (mean±sdv).

	1DCNN		RF		DNN	
	Acc	F1	Acc	F1	Acc	F1
Ori	0.55±0.05	0.44±0.05	0.59±0.04	0.44±0.05	0.47±0.08	0.48±0.04
FS	0.59±0.04	0.40±0.13	0.59±0.04	0.44±0.05	0.40±0.10	0.44±0.06
MS	0.45±0.06	0.35±0.08	0.38±0.12	0.27±0.10	0.39±0.12	0.31±0.10
SM	0.57±0.04	0.46±0.06	0.58±0.04	0.48±0.06	0.36±0.13	0.28±0.11
DA	0.51±0.05	0.47±0.05	0.50±0.07	0.66±0.05	0.52±0.05	0.48±0.05
G1	0.65±0.08	0.63±0.08	0.67±0.03	0.45±0.05	0.59±0.14	0.65±0.07
G5	0.66±0.10	0.66±0.09	0.62±0.07	0.41±0.07	0.64±0.07	0.65±0.05
G20	0.64±0.09	0.65±0.08	0.63±0.06	0.41±0.05	0.64±0.15	0.61±0.09
G100	0.65±0.11	0.63±0.12	0.61±0.08	0.40±0.07	0.67±0.14	0.66±0.13

**Table 8:** Comparison result of three classifiers for BRCA (mean±sdv).

	1DCNN		RF		DNN	
	Acc	F1	Acc	F1	Acc	F1
Ori	0.50±0.05	0.39±0.13	0.58±0.03	0.43±0.04	0.46±0.09	0.45±0.03
FS	0.58±0.03	0.43±0.03	0.58±0.02	0.43±0.03	0.36±0.06	0.44±0.02
MS	0.50±0.11	0.37±0.12	0.48±0.06	0.35±0.05	0.38±0.11	0.30±0.09
SM	0.53±0.02	0.44±0.03	0.56±0.03	0.49±0.04	0.35±0.13	0.28±0.14
DA	0.52±0.07	0.44±0.02	0.46±0.08	0.66±0.02	0.48±0.06	0.45±0.03
G1	0.62±0.08	0.60±0.07	0.63±0.07	0.44±0.07	0.68±0.05	0.66±0.04
G5	0.66±0.10	0.60±0.07	0.63±0.08	0.43±0.05	0.71±0.08	0.69±0.05
G20	0.64±0.06	0.60±0.05	0.64±0.08	0.43±0.06	0.68±0.06	0.62±0.09
G100	0.63±0.06	0.62±0.05	0.64±0.09	0.44±0.05	0.70±0.06	0.57±0.11

**Table 9:** Comparison result of three classifiers for THCA (mean±sdv).

	1DCNN		RF		DNN	
	Acc	F1	Acc	F1	Acc	F1
Ori	0.56±0.04	0.38±0.12	0.58±0.04	0.47±0.04	0.43±0.12	0.49±0.04
FS	0.56±0.02	0.39±0.11	0.57±0.03	0.37±0.05	0.38±0.12	0.38±0.07
MS	0.56±0.07	0.45±0.07	0.51±0.04	0.38±0.04	0.45±0.09	0.37±0.10
SM	0.54±0.03	0.47±0.05	0.55±0.04	0.48±0.05	0.56±0.07	0.44±0.08
DA	0.50±0.04	0.43±0.05	0.47±0.09	0.65±0.06	0.49±0.06	0.47±0.05
G1	0.65±0.16	0.62±0.18	0.60±0.08	0.41±0.08	0.70±0.14	0.61±0.22
G5	0.67±0.10	0.67±0.08	0.55±0.10	0.39±0.07	0.65±0.14	0.64±0.13
G20	0.65±0.13	0.62±0.16	0.54±0.12	0.39±0.08	0.69±0.13	0.57±0.19
G100	0.67±0.13	0.61±0.18	0.54±0.12	0.40±0.08	0.67±0.15	0.63±0.17

**Table 10:** Comparison result of three classifiers for ESCA (mean±sdv).

	1DCNN		RF		DNN	
	Acc	F1	Acc	F1	Acc	F1
Ori	0.43±0.05	0.30±0.06	0.24±0.04	0.25±0.06	0.34±0.05	0.35±0.03
FS	0.37±0.04	0.33±0.05	0.22±0.03	0.23±0.05	0.31±0.06	0.32±0.06
MS	0.44±0.03	0.34±0.06	0.23±0.08	0.24±0.07	0.42±0.30	0.43±0.15
SM	0.36±0.03	0.29±0.07	0.21±0.05	0.21±0.03	0.41±0.06	0.42±0.04
DA	0.38±0.04	0.33±0.06	0.19±0.08	0.22±0.06	0.38±0.03	0.36±0.05
G1	0.46±0.08	0.34±0.08	0.57±0.07	0.52±0.10	0.45±0.12	0.46±0.06
G5	0.65±0.10	0.59±0.10	0.57±0.09	0.51±0.13	0.61±0.11	0.60±0.10
G20	0.65±0.08	0.59±0.09	0.57±0.07	0.52±0.11	0.54±0.14	0.63±0.11
G100	0.61±0.13	0.56±0.13	0.58±0.07	0.53±0.11	0.56±0.11	0.58±0.13

**Table 11:** Comparison result of three classifiers for KICH (mean±sdv).

	1DCNN		RF		DNN	
	Acc	F1	Acc	F1	Acc	F1
Ori	0.34±0.11	0.27±0.10	0.22±0.06	0.29±0.07	0.31±0.03	0.30±0.12
FS	0.50±0.08	0.46±0.07	0.18±0.09	0.24±0.10	0.27±0.11	0.24±0.09
MS	0.41±0.05	0.40±0.09	0.21±0.08	0.22±0.09	0.36±0.06	0.34±0.08
SM	0.39±0.12	0.38±0.14	0.20±0.11	0.21±0.08	0.34±0.09	0.33±0.06
DA	0.36±0.04	0.36±0.06	0.35±0.07	0.43±0.10	0.32±0.11	0.32±0.15
G1	0.44±0.08	0.35±0.09	0.43±0.11	0.38±0.12	0.40±0.11	0.36±0.11
G5	0.41±0.10	0.35±0.11	0.46±0.09	0.43±0.11	0.41±0.06	0.37±0.08
G20	0.42±0.11	0.35±0.10	0.46±0.10	0.42±0.12	0.40±0.06	0.38±0.04
G100	0.43±0.11	0.36±0.11	0.47±0.08	0.44±0.10	0.41±0.11	0.35±0.09

**Table 12:** Comparison result of three classifiers for LIHC (mean±sdv).

	1DCNN		RF		DNN	
	Acc	F1	Acc	F1	Acc	F1
Ori	0.43±0.07	0.35±0.06	0.29±0.03	0.30±0.06	0.42±0.11	0.42±0.04
FS	0.46±0.06	0.44±0.06	0.28±0.0	0.28±0.04	0.39±0.03	0.40±0.03
MS	0.44±0.05	0.46±0.06	0.30±0.11	0.33±0.09	0.36±0.06	0.36±0.11
SM	0.43±0.06	0.42±0.08	0.22±0.08	0.28±0.06	0.37±0.08	0.36±0.05
DA	0.43±0.07	0.41±0.04	0.26±0.11	0.22±0.08	0.33±0.05	0.32±0.06
G1	0.50±0.06	0.46±0.05	0.44±0.09	0.39±0.10	0.44±0.08	0.42±0.03
G5	0.49±0.07	0.46±0.06	0.45±0.96	0.41±0.11	0.46±0.06	0.44±0.08
G20	0.48±0.04	0.46±0.03	0.46±0.09	0.40±0.11	0.44±0.07	0.42±0.06
G100	0.50±0.06	0.48±0.05	0.47±0.10	0.41±0.11	0.45±0.11	0.42±0.09

**Table 13:** Comparison result of three classifiers for LUSC (mean±sdv).

	1DCNN		RF		DNN	
	Acc	F1	Acc	F1	Acc	F1
Ori	0.44±0.03	0.39±0.05	0.28±0.02	0.33±0.04	0.45±0.13	0.45±0.06
FS	0.45±0.04	0.43±0.04	0.24±0.03	0.28±0.03	0.44±0.06	0.42±0.02
MS	0.41±0.08	0.42±0.09	0.26±0.10	0.27±0.06	0.39±0.13	0.40±0.12
SM	0.42±0.03	0.42±0.07	0.24±0.05	0.24±0.07	0.37±0.10	0.38±0.14
DA	0.41±0.06	0.41±0.08	0.20±0.09	0.21±0.04	0.36±0.11	0.38±0.11
G1	0.57±0.09	0.48±0.14	0.61±0.02	0.58±0.02	0.55±0.11	0.54±0.09
G5	0.59±0.10	0.51±0.16	0.62±0.05	0.58±0.07	0.57±0.09	0.55±0.09
G20	0.60±0.09	0.53±0.16	0.61±0.07	0.56±0.10	0.55±0.11	0.53±0.11
G100	0.58±0.09	0.50±0.15	0.60±0.05	0.55±0.08	0.57±0.09	0.53±0.12

**Fig 4:**
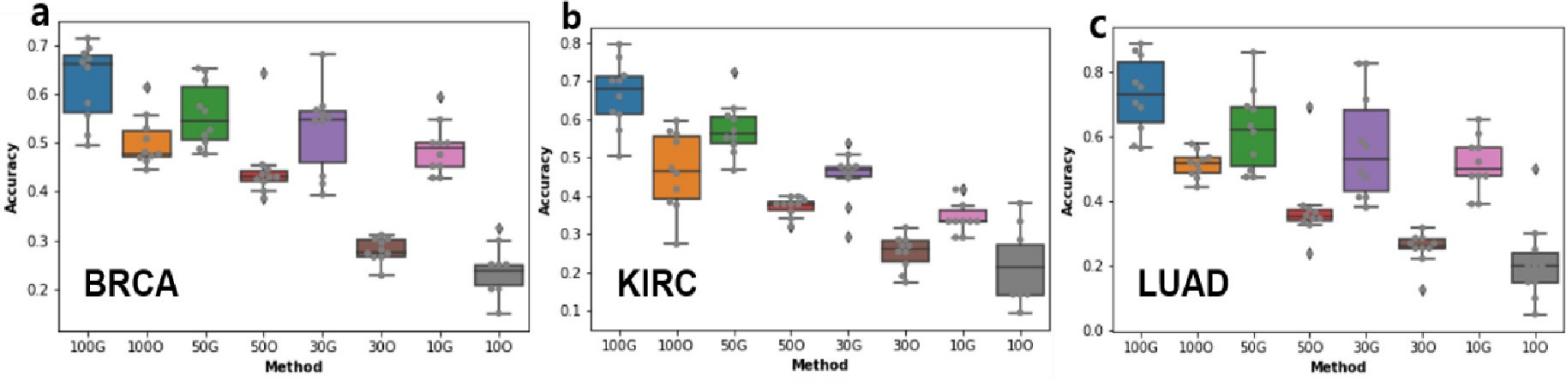
Classification accuracy for randomly sampled data. 100G, 50G, 30G and 10G indicate classification accuracies using 5 times augmented data from 100%, 50%, 30%, and 10% randomly selected samples, respectively. 100O, 50O, 30O, and 10O indicate classification accuracies using 100%, 50%, 30%, and 10% randomly selected samples (same as those for 100G/50G/30G/10G), respectively. Classification algorithm used is 1DCNN, and each random sampling was performed 10 times.

**Fig 5:**
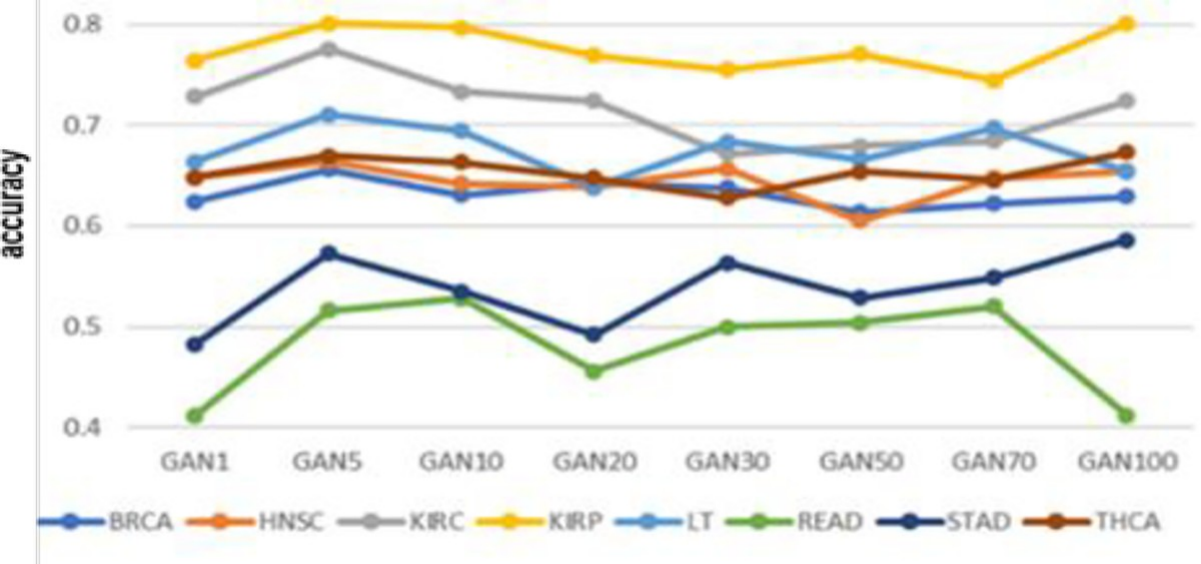
Optimal augmentation fold. Each fold was repeated 10 times.

Next, we examined whether the proposed sample augmentation method is effective for datasets with small samples. We used whole samples and randomly selected 50%, 30%, and 10% of samples from BRCA, LUAD, and KIRC datasets, and applied 1DCNN. The results are shown as 100O, 50O, 30O, and 10O in (Fig 4). We next expanded the sampled datasets 5 times (GAN5) and applied 1DCNN. The results are shown as 100G, 50G, 30G, and 10G in (Fig 4). We can see that reducing the number of samples lowers the classification accuracy; however, accuracies are much higher when samples are augmented. More importantly, we can see that the decrease in accuracy is generally smaller when samples are augmented. These results imply that the proposed method is effective for small datasets. Lastly we performed experiments to determine the optimal fold for sample augmentation. We compared classification accuracies from samples augmented by 1, 5, 10, 20, 30, 50, 70, and 100 fold. The results are shown in (Fig 5). In general, we can conclude that the optimal folds differ for different cancer types; however, we can observe that 5 fold (GAN5) demonstrates generally good results.

### Analysis of selected genes

We additionally verified that genes were selected properly, in this section. Eight published studies [25–32] of TCGA included hyper-mutated genes and non-hyper-mutated genes. Among those genes, we selected significantly mutated genes for each cancer using MutSig [33, 34] and MuSiC [35], and summarized them in Table 14.

**Table 14:** Significantly mutated gene selected by TCGA.

Type	Gene list
BRCA	AKT1, CDH1, CDKN1B, GATA3, MAP3K1, PIK3CA, RYR2, TBX3, TP53, MLL3, MAP2K4, RUNX1, PTEN, PIK3R1, CBFB, TBL1XR1, NCOR1, CTCF, ZFP36L1, GPS2, SF3B1, USH2A, RPGR, RB1, AFF2, NF1, PTPN22, PTPRD, OR6A2, HIST1H2BC, GPR32, CLEC19A, CCND3, SEPT13, DCAF4L2
READ	APC, KRAS, TP53, PIK3CA, FBXW7, CSMD3, TNN, NAV3, SMAD4, EPHA3, MAP2K7, EPHB6, PTEN, ADAMTSL3, GUCY1A2, SMAD2, OR51E1, LAMA1, C10orf137, TCF7L2, ADAMTS18, FBN2, TGFBR2, SEC8L1, RET, KIAA2022, MMP2, GNAS, STAB1, AGC1
THCA	BRAF, NRAS, HRAS, E1F1AX, PPM1D, KRAS, CHEK2, TP53, ARID1B, MLL, BDP1, PTEN, TG, ZFHX3, ATM, RB1, TSHR, EZH1, MEN1, CDH4, SPOP, MLL3, APC, NF1
HNSC	CDKN2A, FAT1, TP53, CASP8, AJUBA, PIK3CA, NOTCH1, KMT2D, NSD1, HLA-A, TGFBR2, HRAS, FBXW7, RB1, PIK3R1, TRAF3, NFE2L2, CUL3, PTEN
KIRC	VHL, PBRM1, SETD2, KDM5C, BAP1, PTEN, MTOR, TP53, PIK3CA
KIRP	BRAF, NRAS, HRAS, E1F1AX, PPM1D, KRAS, CHEK2, TP53, ARID1B, MLL, BDP1, PTEN, TG, ZFHX3, ATM, RB1, TSHR, EZH1, MEN1, CDH4, SPOP, MLL3, APC, NF1
LUAD	TP53, KRAS, KEAP1, STK11, EGFR, NF1, BRAF, SETD2, RBM10, MGA, MET, ARID1A, PIK3CA, SMARCA4, RB1, CDKN2A, U2AF1, RIT1
STAD	TP53, CDH1, SMAD4, PIK3CA, RHOA, ARID1A, KRAS, MUC6, APC, BCOR, EYA4, BNC2, RNF43, ABCA10, CTNNB1, MACF1, SMAD2, SOHLH2, RASA1, FAM46D, PLB1, CNGA4, EIF2C4, ERBB2, PTPRC

**Fig 6:**
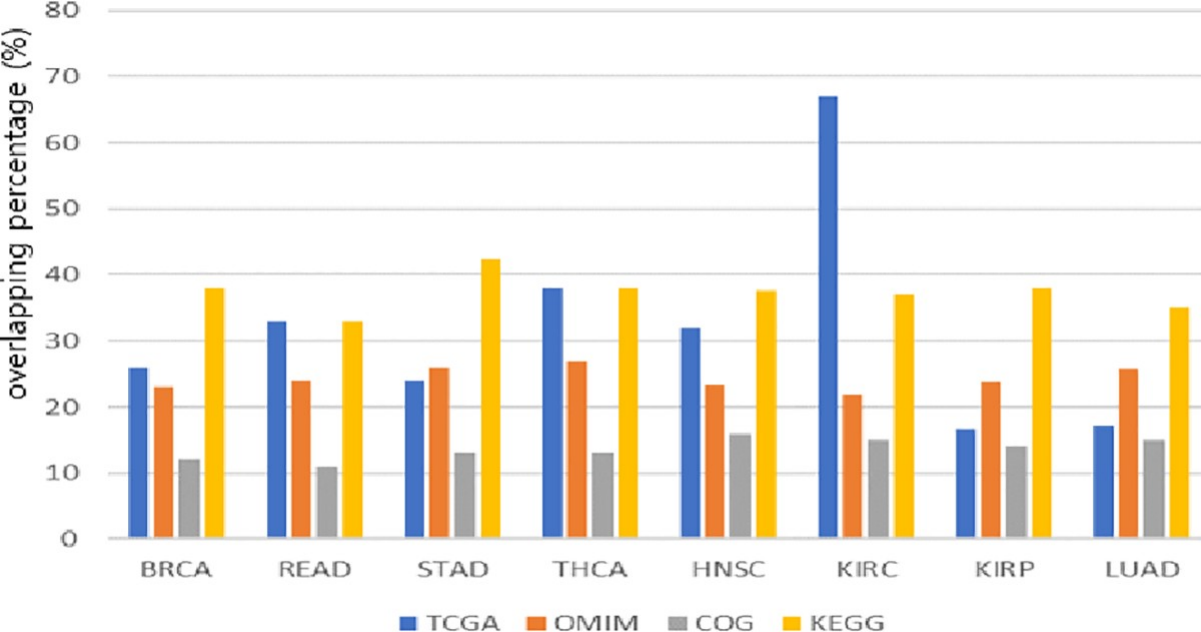
Association of pathways with diseases of selected genes.

Selected genes in the current study (S1 Table) frequently harbor those mutations. For example, 17% (KIRP), 17% (LUAD), 24% (STAD), 26% (BRCA), 32% (HNSC), 33% (READ), 38% (THCA), and 67% (KIRC) of selected genes were overlapped with genes in Table 14, as shown in (Fig 6).

KIRC matches six out of nine genes. VHL and PBRM1 are major genes that cause mutations in more than 40% of clear cell renal cell carcinoma, and SETD2 and PTEN, which are quite frequent, are genes that cause both copy number loss and mutation. The BRAF gene of THCA is the most important gene with 60% missense mutation and more than 2% fusion, and includes a list of most oncogenes such as NRAS, TP53, PTEN, and RB1.

Selected genes are also overlapped with genes in the Online Mendelian Inheritance in Man (OMIM) database (23–27%, [36]), the Clusters of Orthologous Groups of proteins (COG) database (about 15, [37]), and the Kyoto Encyclopedia of Genes and Genomes (KEGG) database (33~43% [38]). We can see that the overlapping percentages of KEGG are the largest in general, which means that a significant number of genes are important genes involved in the pathway. The PI(3)K/AKT/MTOR pathway (altered in 28% of tumors) has been shown to be important in KIRC by papers published by TCGA, and genes in S1 Table match the PI3K-AKT pathway with p-value 0.026. It contains most of the upstream genes of the AKT pathway, for example PIK3CA, PTEN, Receptor Tyrosine Kinase (RTK)-related genes (EPHB, PDGFR) and Integrin Subunit (ITG)-related genes (ITGA7, ITGA9, ITGA11, ITGB1BP, LABA, LAMB, THBS).

A Warburg effect-like state achieved through downregulation of AMP-activated kinase (AMPK) and upregulation of acetyl-CoA carboxylase (ACC) has also been shown to be important in cancers. Among the genes in S1 Table, ATP binding transporter pathway genes (ABCA, ABCB, ABCC, CFTR), extracellular matrix receptor (ECM) genes (COL1, COL4, COL5, COL6, COL11, ITGA, LAMA, LAMB, LAMC, THBS, TNC, TNN, TNXB, AGRN), and Krebs cycle genes (ACAT, ACOX, ACSBG, ADH1, CAMK1, CAMK2G, ECI, FBP, PFKFB, PDHA, SIRT3, SLC2A) were found.

## Discussion

We noted that both GAN5 and GAN20 show good results in that the error bars are generally narrower in most of the carcinomas than those of GAN1, in (Fig 3). This observation indirectly demonstrates that increasing the number of samples leads to increased classification accuracy. In addition, it can be confirmed in Tables 2–13 that the 1DCNN classification method was excellent in both accuracy and F1 score. In Jian Liu’s paper [17], Sample Expansion-Based 1DCNN (SE1DCNN), a method of obtaining a large number of samples through multiple, partially corrupted inputs, improved accuracy by 1–9% compared to the method using only 1DCNN. In addition, Sample Expansion using the Sample Expansion-Based SAE (SESAE) method improved accuracy by 2–17% compared to using only the Stacked Autoencoder (SAE). It was confirmed that when a good sample augmentation method and a good classification model are combined, there is better improvement of performance, and development of good combined models is always required.

The optimal number of samples differs for different cancer types, as observed in (Fig 5). Our model used one hidden layer with 256 neurons, which is the most suitable size for an imbalanced data set, according to the previous study [23]. However, further study is needed of the remaining five options (256/512/102, 256/512, 128/256/512, 128/256, and 128). In addition to these results, optimization of the hyperparameters (such as learning rate, epochs, cost function, and hidden layer unit) used in our GAN model, need additional work.

In addition to the DNA mutation data used for feature selection in this study, various combinations of more omics data such as mRNA, DNA methylation, and miRNA data can be used to further increase the classification accuracy. Application of those data combinations will be the focus of our follow-up work. Moreover, various recently developed deep generative models such as DCGAN, cycleGAN, and Variational Autoencoder, could be explored for more accurate classification, which could be our future study.

## Conclusions

In this paper, we proposed the sample augmented method using GANs, and showed that augmented samples significantly increased the classification accuracy of cancer stages. In particular, we were able to confirm that the proposed method is efficient for a dataset with small number of samples. Therefore, the proposed sample augmentation method can be applied for other purposes, such as prognostic prediction or cancer classification.

● Advantages
  - The proposed method can generate additional data samples more accurately, which can increase the accuracy of cancer-stage prediction.
  - The proposed method is generally applied to other types of mRNA expression data of which the aim is different from cancer-stage prediction.
● Disadvantages
  - If the number of features is large, the learning time is significantly slower than with other machine learning approaches such as random forest or gradient boosting.

## Supporting information

S1 Table List of all genes selected by features selection. (XLSX)

S2 Table Results of t-test and friedman test. (XLSX)

S3 Table The result of newly added cancers and random forest. (XLSX)

S1 File (DOCX)

## Abbreviations

TCGA: The Cancer Genome Atlas
TNM: Tumor, Node, and Metastasis
SMOTE: Synthetic Minority Oversampling Technique
GANs: Generative Adversarial Networks
1DCNN: 1-Dimensional Convolutional Neural Networks
