# Supplementary material for: Increasing prediction accuracy of pathogenic staging by sample augmentation with a GAN
Source: PLoS One. 2021 Apr 27;16(4):e0250458. doi: 10.1371/journal.pone.0250458 (PMC8078779; doi:10.1371/journal.pone.0250458)
Supplement: S1 File — (DOCX) [file pone.0250458.s004.docx]

**Data Availability Statement:**

Source programs to generate the results is available at <https://github.com/narrowpath/SampleAugmentationWithGAN> or [DOI 10.17605/OSF.IO/MG8RW](https://osf.io/MG8RW/)

We downloaded mRNA(RNASeqData) and DNA mutation(SomaticMutationData) data of twelve cancer types using [TCGA-assembler2](http://www.compgenome.org/TCGA-Assembler/) (http://www.compgenome.org/TCGA-Assembler/) from the [TCGA database](https://doi.org/10.5114/wo.2014.47136). The twelve types of cancer included stomach adenocarcinoma(STAD), breast invasive carcinoma(BRCA), head and neck squamous cell carcinoma(HNSC), kidney renal clear cell carcinoma(KIRC), kidney renal papillary cell carcinoma(KIRP), lung adenocarcinoma(LUAD), thyroid carcinoma(THCA), rectum adenocarcinoma(READ), esophageal carcinoma(ESCA), kidney chromophobe(KICH), liver hepatocellular carcinoma(LIHC), and lung squamous cell carcinoma(LUSC).
